# Evaluation of a photographic method to measure dental angulation

**DOI:** 10.1590/2176-9451.19.2.084-089.oar

**Published:** 2014

**Authors:** Jordana Rodrigues Amorim, Diogo de Vasconcelos Macedo, David Normando

**Affiliations:** 1 Student at the specialization course in Preventive Orthodontics, Hospital for Rehabilitation of Craniofacial Anomalies/USP; 2 Degree in Dentistry, Federal University of Pará (UFPA); 3 Adjunct professor, Federal University of Pará (UFPA)

**Keywords:** Dental crown, Dental photography, Evaluation

## Abstract

**Objective:**

To analyze the reliability and reproducibility of a simplified method for analysis of dental angulation using digital photos of plaster dental casts.

**Methods:**

Digital and standardized photographs of plaster casts were performed and posteriorly imported to an angle reading graphic program in order to have measurements obtained. Such procedures were repeated to evaluate the random error and to analyze reproducibility through intraclass correlation. The sample consisted of 12 individuals (six male and six female) with full permanent dentition orthodontically untreated. The analyses were bilaterally carried out, and generated 24 measurements.

**Results:**

The random error showed variation of 0.77 to 2.55 degrees for teeth angulation. The statistical analysis revealed that the method presents excellent reproducibility (p < 0.0001) for all teeth, except for the upper premolars. In spite of that, it is still considered statistically significant (p < 0.001).

**Conclusion:**

The proposed method presents enough reliability that justifies its use in the development of scientific research as well as in clinical practice.

## Abstract (pt)

**Objetivo:**

analisar a confiabilidade e reprodutibilidade de um método simplificado para análise da angulação dentária que faz uso de fotografias digitalizadas de modelos de gesso.

**Métodos:**

foram realizadas fotografias digitalizadas e padronizadas de modelos de gesso, posteriormente transportadas para um programa gráfico de leitura de ângulos, para a obtenção das medidas. Tais procedimentos foram repetidos para avaliação do erro do método casual e para a análise da reprodutibilidade por meio da Correlação Intraclasse. A amostra constituiu-se de 12 indivíduos com dentição permanente completa e não tratados ortodonticamente, sendo seis do sexo masculino e seis do feminino. As análises foram feitas bilateralmente, gerando 24 medidas.

**Resultados:**

o erro casual mostrou uma variação de 0,77 a 2,55º para a angulação dos dentes. A análise estatística revelou que o método apresenta uma excelente reprodutibilidade (r = 0,65 - 0,91; p < 0,0001) para todos os dentes, exceto para os pré-molares superiores, mas ainda assim estatisticamente significativa (p < 0,001).

**Conclusão:**

o método proposto apresenta confiabilidade suficiente para justificar seu uso no desenvolvimento de pesquisas científicas, bem como na prática clínica.

## INTRODUCTION

Knowledge on mesiodistal angulation of dental crowns became more prominent after the study described by Andrews in 1972, regarding the "Six keys for an optimal occlusion,"^1^ among which the second key states that a tooth correctly angulated in the arch is one of the criteria used to achieve functional occlusion. In this study, the angulations of the dental crowns were determined by measuring the angle formed between the buccal axis of the clinical crown with a line perpendicular to the occlusal plane, on the specimens previously cut out in the center of the clinical crown, using a plastic protractor.

Although undoubtedly important, Andrews' work was applied to only one sample of American individuals and on the reproduction of his methods, it was observed difficulty regarding the accuracy on the positioning of rulers and protractor, thus, generating doubts and high variance of results.^3^ Moreover, the cut out of specimens mentioned in that method proves to be inconvenient due to the amount of work and clinical time that is spent, in addition to the discard of orthodontic models.

From the values obtained by Andrews' study, the means of angulations for each dental crown were established. It is possible, however, to assert that dental angulations do not always follow a constant pattern, so that there might be variability on angular values according to the individual characteristics of each patient, considering different cases of malocclusion. Thus, studies that engage in learning and evaluating the ideal mesiodistal positioning become undoubtedly relevant.

A device projected specifically to measure the angulations and inclinations of the dental crowns in plaster casts has been recently presented.^3^ With the development of this device, which consists of an alteration in the original methods of Andrews, it was possible to establish mean values of angulation and inclinations of dental crowns for Brazilian individuals with normal occlusion. However, this methods involve a device that is not available in the market, thus, requiring customized production.

Computed tomography (CT) can be useful to assess dental inclinations and angulations,^4^ allowing great progress to researches involving tooth positioning and, also, to the individualization of orthodontic treatment. However, since this method is relatively new and still needs further studies that prove its efficiency and reliability, it must be used with reservations. Furthermore, the high costs of CT scans and the risks inherent to its radiation are some of the disadvantages of this technique.

Therefore, there is a need for methods of simple application that allow the orthodontist to identify and quantify, in a reliable manner, the mesiodistal positioning of the dental crowns, as well as the natural compensation existing in many patients with malocclusion. Thus, the objective of this study is to evaluate the reliability of a photographic method previously described, which, however, had been limited to analyzing canine angulation.^5,6^ In the present study, this method was used to quickly and simply measure the angulations of all teeth anteriorly positioned in relation to the permanent first molar.

## MATERIAL AND METHODS

This study was approved by the Institutional Review Board of the Institute of Health Sciences of the Federal University of Pará (ICS/UFPA), under protocol number 154-09 and by the National Committee of Ethics in Research (CONEP), under number 25000.066559/2010-11, report number 462/2010.

The sample comprised 12 individuals with permanent dentition and with natural normal occlusion, of which six were males and six females, with mean age of 13.7 years old. The angulations of the first molars, premolars, canines and incisors were bilaterally obtained, in both arches of the initial casts of the selected individuals, thus generating 24 measurements for each tooth, according to the method previously described.^5,6^

**Figure 1:**
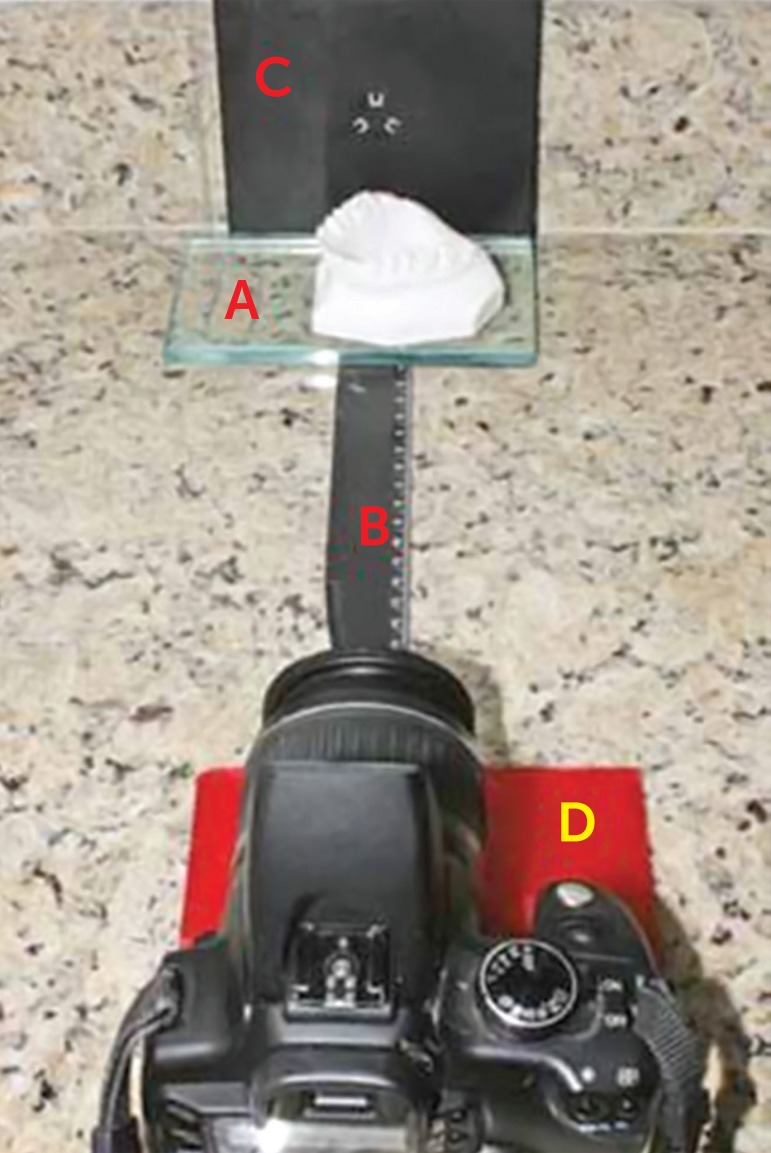
Method used for standardization of the photographic takes of the plaster molds: A= 10-mm-thick glass plate; B= 20-cm millimeter ruler; C= black plastic plate with mark indicating the center of the object (the back of a CD-ROM case), D= red wax plate.

**Figure 2:**
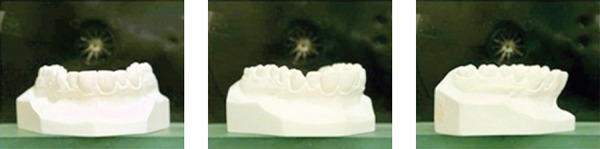
Plaster molds used in the sampling.

In this method,^5,6^ the specimens were positioned on a glass plate, at a distance of 20 cm from the photographic lens. A black device with a mark in the center was placed in the background of each specimen, and used as reference to centralize the group of teeth that would be examined. The camera lens was placed against a red wax plate in order to optimize the direction of the lens (Fig 1). Five photographs were taken per arch: two lateral - one in each hemi arch - centralizing the second premolar to the mark in the background, to measure the angulation of the first molar and the premolars; two diagonal, having the line between the canine and the lateral as the central point for the bilateral measurement of these teeth; and one frontal for the measurement of the central incisors (Fig 2).

Ten photographs were taken per individual, totalizing 120 photographs which were then imported to a computer software (Paint Microsoft^®^, Microsoft Corporation, Redmond, USA) in order to have the occlusal plane drawn. Posteriorly, the images were imported to a graphical computer software (Image Tool^®^ - www.imagetool.com, Image Tool Software, USA), in order to measure teeth angulation.

In both lateral and diagonal photographs, the line corresponding to the occlusal plane was traced from the incisal surface of the central incisors to the mesiobuccal cusp of the permanent first molar. On the frontal photographs, this line was traced touching the tip of the cusp of the canines, as proposed in previously published researches.^5,6^

**Figure 3:**
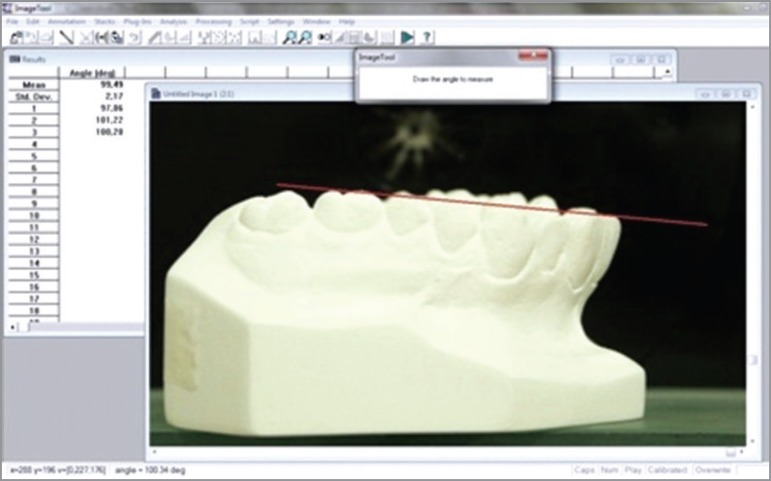
Photograph of the plaster mold exported to the graphic software used to obtain the measurements of the dental angulations.

Posteriorly, through Image Tool^®^, the long axis of the canine's clinical crown was traced and, from the intersection of these two lines (occlusal plane and long axis), the value of angulation of the clinical crown of the plaster cast was obtained (Fig 3). As for random error analysis, the hemi-arches of the initial plaster molds of all patients were photographed again after 7 days and all the aforementioned steps were repeated until new measurements of the dental angulation were obtained. The random error was calculated according to Dahlberg's formula . The analysis of reproducibility was performed by the test of intraclass correlation, both with a level of reliability of 95%.

## RESULTS

The results revealed a minor random error, less than one degree, except for the upper molars (2.55 degrees). With regard to the analysis of reproducibility (intraclass correlation), the statistical analysis revealed an excellent reproducibility of the method (p < 0.0001), except for the upper premolars, of which reproducibility was classified as being from regular to good (r = 0.65 and 0.71, p < 0.001) (Table 1). The results also revealed that the difference of the angulation means was relatively minor and less than one degree, except for the upper first molars (2.29 degrees) (Table 2).

**Table 1:** Upper and lower teeth - Random error, method reproducibility (intraclass correlation) and analysis of normality of the distribution of values.

Tooth	Random error	Intraclass correlation (r)	P value	Reproducibility
U6	2.55	0.76	P < 0.0001	Excellent
U5	1.98	0.65	P = 0.0003	Regular to good
U4	2.35	0.71	P < 0.0001	Regular to good
U3	1.84	0.91	P < 0.0001	Excellent
U2	1.12	0.93	P < 0.0001	Excellent
U1	0.77	0.92	P < 0.0001	Excellent
L6	1.58	0.84	P < 0.0001	Excellent
L5	1.86	0.84	P < 0.0001	Excellent
L4	1.62	0.88	P < 0.0001	Excellent
L3	1.57	0.90	P < 0.0001	Excellent
L2	1.55	0.91	P < 0.0001	Excellent
L1	1.06	0.78	P < 0.0001	Excellent

**Table 2:** Upper and lower teeth - Angulation means and difference means.

Teeth	Mean – T_1_	Mean – T_2_	Difference means
U6	89.69	91.98	-2.29
U5	90.02	89.99	0.03
U4	89.48	89.69	-0.22
U3	92.46	91.47	0.98
U2	100.2	99.6	0.6
U1	96.47	96.24	0.23
L6	95.56	95.92	-0.36
L5	92.82	93.15	-0.33
L4	88.61	88.94	-0.325
L3	94.57	93.52	1.06
L2	91.44	90.51	0.94
L1	89.93	89.77	0.16

## DISCUSSION

The degree of reliability of measurements taken in specimens has not yet been properly evaluated, perhaps because, originally, it is considered a direct method. However, the changes used in the present study showed that the method used to measure the angulations of dental crowns is greatly reproducible, presenting a random error equal to or less than 2.55 degrees, in addition to being very simple and avoiding the need for detrition of the specimens.

Some methods have been described in the literature for the evaluation of teeth angulation, similarly to the method proposed by Andrews, which uses measurements directly taken on the specimens through a plastic protractor,^1^ and others that involve advanced technological resources, such as the use of computed tomography.^4^ Other methods also describe the use of devices specially developed for this purpose.^3,7,8^ It is natural, however, that each one of the described methods present inherent difficulties, such as certain imprecision on the positioning of the measuring tool,^1^ cut out and discard of the plaster molds,^1^ high costs,^4^ radiation risks^4^ and devices that are not available in the market and, for this reason, have to be customized.^3,7,8^

With the technological progress, modern computer software allow reliable analysis of images at low costs. Thus, for the present study, a computed graphical program capable of accurately reading the teeth angulations from the standardized digital photographs of the plaster molds was used.

The method proposed presents differences in relation to Andrews' original proposal. First, the occlusal plane, that, in this study, was represented by a line that connected the incisal surface of the incisors to the tip of the mesiobuccal cusp of the first molar and, on the frontal photographs, a line that touched the tip of the cusp of the canines. This plane is not always parallel to Andrews' plane, especially in cases of malocclusion.

The values found by Andrews and described as normal were very important factors for the development of the Straight-Wire technique, which aimed to individualize the appliance according to patient's orthodontic needs.^2^ This pre-programmed appliance uses brackets individually built for each tooth: to these brackets is conferred the information about the ideal mesiodistal and buccolingual position that each element must achieve by the end of the orthodontic treatment. However, the occlusal and skeletal characteristics of each patient are unique, which hinders the creation of a rigid protocol for the detailing of the cases. Since then, many orthodontists began to individualize brackets angulation according to their clinical experience, due to the morphological variations inherent to the dentofacial complex, being most of these modifications introduced without any scientific support.

What seems to be clinical evidence based on the experience of each professional, needs scientific evaluation that support or not the changes inserted on orthodontic appliances used with the objective of individualizing the cases. Methods of simple application, which allow the orthodontist to identify the presence of existing natural compensations, or even to reliably quantify them, would allow the clinician to extend the use of this concept in a scientifically adequate way.

The challenge of developing accessible methodological procedures is the main factor responsible for the lack of studies aimed at discovering the buccal-lingual and mesiodistal positioning of each dental element in the arch. Thus, a method that allows analysis of the dental angulation in a quick and simple way through materials easily available and software of free access represents a great resource to clinicians.

The method for analysis of the dental angulation described and tested in the present study had been previously used to compare the angulation of canines in individuals with Class I and III malocclusion,^5^ as well as to analyze the correlation between the angulation of canines and the inclination of incisors.^6^ In both studies, the random error and the systematic error were evaluated and yielded results based on which it can be concluded that the method is considered valid. However, both authors only applied and evaluated this method in anterior teeth and in lateral photographs. It is worth noting that no studies have been yet conducted to prove the reproducibility and reliability of the proposed method when applied to analyze the mesiodistal angulations of the crowns of all teeth anterior to the permanent first molar.

By using and testing the new method proposed in the present study, it is reasonable to conclude that the suggested method presents excellent reproducibility (P < 0.0001), agreeing with the results described by other authors,^5,6^ except for the upper premolars that presented a reproducibility classified as being from regular to good (P < 0.001), but yet statistically significant.

The lowest reproducibility found in the measurements performed on upper premolars possibly occurred due to the shorter length of the clinical crown of these teeth, since these teeth present a shorter cervico-occlusal distance the more posterior they are. Once premolars do not present reference lines on the buccal surfaces, as molars do, in addition to being shorter teeth, it is possible to find greater variance on the marking of the long axis of the crown and consequent distortion on the obtained results.

## CONCLUSION

Based on the analysis of the results of this study, it is reasonable to conclude that the method described herein presents enough reliability to justify its use both in clinical practice and as auxiliary means on the development of scientific research that focus on the evaluation of the angulations of dental crowns. Moreover, it presents excellent reproducibility, without any difference between the two measurements performed, and with random error relatively small, it achieves the initial proposal of reducing the time that is necessary to take the measurements. Furthermore, it is easy to be executed by the clinician and also allows the preservation of the plaster casts , important objects of orthodontic documentation of the patient.
